
JOURNAL INFORMATION
==============================
NLM Title Abbreviation: Med Chir Rev
NLM Journal ID: 0034617

The Medico-Chirurgical Review

Publisher: Biomedical Journal Digitization Project

ARTICLE INFORMATION
==============================
PMCID: PMC4966246
PMID: 29917618
Article version: 1
Subjects: Articles

Periscope, or Circumspective Review

Print publication date: 1828 Oct 1
Volume: 9
Issue: 18
First page: 433
Last page: 444
1828 Jul
License: This work is free of known copyright restrictions. For more information, please see PMC Back Issue Digitization.
License URL: https://creativecommons.org/publicdomain/mark/1.0/

==============================

### Periscope;

OR,

### CIRCUMSPECTIVE REVIEW.

"Ore trahit quodcunque potest, atque addit acervo."

### 1. London University—Medical Education.

Nam vehementer intererat vestra, qui patres estis, liberos vestros hic notissimum discere. Ubi enim aut jucundius morarentur, quam in patria? Aut pudicius continerentur, quam sub oculis parentum? Aut minore sumtu, quam domi?—*Plin. Ep. lib.* iv.

Our descendants will anxiously inquire the causes that could have prevented the formation of a University in the metropolis of this great country before the year 1828! They will be astonished to learn these causes—if, indeed, the arguments against a metropolitan university should go down to posterity, which we much doubt. Universal experience is against the arguments in question. How would a Frenchman stare, if he were told that the University of Paris would be much better located at Tours or Moulins! How would honest Sawney smile, if any *Southron* assured him that literature, science, and art, would be much better taught and studied at Cromarty or Kilmarnock, than in Auld Reekie! The proposition is absurd; and we are convinced that no man, unbiassed (we might say, not blinded) by religious or political prejudice, ever undertook the advocacy of such a proposition—save and except the hireling sophist who writes for pay, and not from principle.

But, admitting that divinity, classics, mathematics, and even law, can be more quietly studied on the banks of the Cam than of the Thames, no one can presume to say that medicine, in all its branches, can be taught in the former locality. As it is with this last department of human knowledge that we have to do, so we will leave the advocacy of the new university to the care of other and abler hands, as far as regards literature and science generally. That the healing art will be greatly benefited by the new institution, we have not the smallest doubt. English medical education is more defective in classical learning, and the accessory or collateral sciences, than in the purely professional part. This is owing, in a great measure, to the want of a University, where the whole circle of literature and science is under one roof. How is a Borough or Bartholomew student to acquire or improve his classical, mathematical, or other extra-professional knowledge? In the new University, where all the medical will be in contact with the other liberal sciences, the student will have neither excuse nor difficulty in expanding his mind, by the cultivation of the collateral branches of human knowledge. If it be said that the medical student will learn no more than he is forced to do by the regulations of his college or company, we reply that the "march of intellect," however that term may be ridiculed or abused, will as effectually compel medical men to keep pace with the general progress and diffusion of knowledge, as custom will force them to wear hats instead of turbans on their heads, or shoes instead of sandals on their feet. The youth of other professions and pursuits are rapidly acquiring the elements of general knowledge—and to think that medical practitioners can keep up a proper intercourse with that world to which they are inferior in general acquirements, is to indulge in a dream from which the dreamer will awake too late! It is this liberal or extra-professional education which makes the Oxford and Cambridge graduate hold his head so high—and no man can deny the advantages which it confers. These advantages, too, will, for a time, be enhanced by the privileges annexed to Oxford and Cambridge, in the three learned professions. But if it be true, and we believe it is, that "knowledge is power," so the actual possession of that knowledge will soon render comparatively valueless the mere seal which indicates the spot where the knowledge is obtained. If Oxford, Cambridge, and other marts of learning have risen and flourished *by degrees*, so will they decay *by degrees*, in obedience to that law which revolves, and for ever will revolve, the wheel of fate—that law which rolled arts, arms, and literature from East to West—from South to North—from Egypt to Greece—from Greece to Italy—and from Italy to the banks of the CAM and the ISIS.

Granting that certain honours and privileges be perpetuated to these later seats of learning, through—

"The boast of heraldry—the pomp of power,"

can it be doubted, for a moment, that a million and a half of people, in this metropolis and its immediate vicinity, rich as they have long been, and thirsty after knowledge, as we hope they will always be, cannot support one UNIVERSITY at least, as well as Edinburgh, Dublin, Glasgow, Paris, Vienna, Berlin, and twenty other capitals, that have not one fiftieth part of its wealth or spirit? It cannot be—it *is not*, doubted, even by the most bigoted adversaries of liberal education.

And are there no advantages accruing to youth from studying in his own country—under the eye and the roof of his parents—and at a comparatively trifling expense? Ask PLINY, and he will tell you, in the motto which is prefixed to this article. Ask COMMON SENSE, which is superior to the authority of a Pliny, or even a Plato. Ask EXPERIENCE what is the cause of the depravity, vice, intemperance, and the thousand acts of licentiousness which reign triumphant at the two great seats of learning in this country? It is the concentrated congregation of youth in two sequestered spots, far from the surveillance of their parents, and with money to spend! What is it but this same concentration which makes, on a small scale, every public seminary for children, a hot-bed of vice? Now we fearlessly maintain that a metropolitan university is infinitely more free from these radical defects than Oxford, Cambridge, or even a public school. The students will be congregated together only during the hours of tuition. These hours past, they will be dispersed in a thousand different directions, to the houses of their parents or guardians, where they can have no intercourse with their fellow students till the hours of study again commence. No man, whether divine or lawyer, can have such an intimate knowledge of human nature as the medical man. We appeal to him, whether or not we have ample foundation for the above observations and reflexions, as far as *moral* circumstances are concerned in the education of youth. We are aware, indeed, that the new university, like all new institutions, will have difficulties to contend with at the beginning—more especially as respects the *clinical* branch of medical instruction. In all other branches, the love of fame alone will attract a host of candidates, of the very first order of talents, and this circumstance will prove a stimulus to each and every medical school in this great metropolis. After the complete organization of this national institution, nothing but *superlative talent* will keep up the private schools of medicine and surgery. And, when a university hospital is established (which will soon be the case) and regular clinical *instruction* (for it hardly deserves that appellation at present) is given—then let the great hospitals of London look out! If the medical officers do not exercise their *heads* as well as their *feet*, they will soon find that the "MARCH of INTELLECT" is not entirely performed by means of the glutei, vasti, and gastrocnemii muscles. The head, the heart, and the hand, must all co-operate in rendering the wards of hospitals productive of a triple or quadruple crop of advantages—health to the sick, instruction to the pupils, reputation to the clinical teacher—and information to the profession at large.*

---

* We have made no allusion to the rival university which is to be established still farther West—report says near St. George's Hospital. We wish it success. It will only render the professors of the primary institution more vigilant and industrious. It is curious, however, that the clerical and aristocratical party which are now setting up a university, declaimed against the necessity, or even the utility of such a seminary! Now it appears that two universities are necessary! So we

In another short article we shall give a sketch of the arrangements in the medical department of the new University, preparatory to its opening in October next.

---

### 2. FATAL DISEASE OF THE SPLEEN.

We should not be surprized if future experience determines that the spleen is a more important organ than is generally supposed. There is little doubt, at all events, that its diseases exercise a very baleful influence on the human constitution. The following case is reported in the last volume of the Transactions of the Swedish Society of Medecine, by M. Westman.

An unmarried woman, 28 years of age, was exposed to cold when in a state of perspiration, by which the latter was checked, and afterwards the menses suppressed. These accidents were followed by colicky pains and swelling of the epigastric region. And to these phenomena succeeded a hæmorrhage from all the natural outlets of the body. This hæmorrhage having ceased, it was observed that the spleen was considerably enlarged. A serous effusion into the cavity of the abdomen next ensued—then the menses re-appeared—became again suppressed—and a new hæmorrhage from all outlets of the body took place in so violent a degree as to destroy the life of the patient. On dissection, the liver was found in a state of atrophy, while the spleen was of enormous size, its parenchymatous structure transformed into a grumous glutinous fluid, enveloping three bony concretions, one of which was two inches and a half in length.

How far the disease of the spleen was the cause of the hæmorrhages, it is not easy to say. There can be little doubt, however, that the checked perspiration and suppressed menstruation played a part in the etiology of the splenic affection.

---

think. We hope to see three or four established before we die. We entirely overlook the little party feelings that are in operation. We contemplate the increased facility for intellectual improvement which must result from these rival institutions.

### 3. DISTRESSING CASE OF NEURALGIA.

[For Consultation.]

The motives which lead us to publish the following most melancholy and terrible case will be readily appreciated. It is with the view (would that we could say HOPE) of eliciting some suggestion that may tend to mitigate the sufferings of an afflicted member of our own profession.

Mentem mortalia tangunt!

The patient is a surgeon, retired from the East India Company's service, and in the 59th year of his age. About nine years ago, a pound of gun-powder (in a cannister) exploded in his left hand, by which the bones of the thumb were fractured, and the soft parts about the palm of the hand much lacerated. The wound was dressed, and the case appeared to be doing well till the 10th day, when the sloughing process took place, which was followed by a profuse hæmorrhage, at first thought to be of venous blood. This was restrained by pressure; but it repeatedly recurred, and proved to be arterial. Several attempts were made to secure the vessel by ligature, but all in vain. Two medical gentlemen (Dr. S. and Mr. C.) advised ligature of the radial artery. The vessel was taken up by the latter gentleman, but unfortunately the radial nerve was included, and, when the ligature was drawn, the patient started up involuntarily from the recumbent to the perpendicular posture, and felt a most dreadful pain dart *from the occiput to the forehead*. This pain was of short duration, but ever since that time he has been subject to head-ache. The hæmorrhage returned the same day, and Dr. S. in hopes of reaching a sound part of the palmar arch, removed the thumb, at its carpal junction. This operation also failed, and hæmorrhage again returned during the night. Being now much reduced, the patient himself determined on amputation, and the operation was accordingly performed the same night. But new misfortunes seemed destined for the unfortunate patient. While the surgeon was in the act of sawing the bones, the assistant let the soft parts fall into the teeth of the saw. "The pain," says the patient, "at this moment, was most exquisite; that produced by the circular incision was pleasure compared with it.

The sensation was as if melted metal were poured into the wound." One or two more strokes of the saw completed the operation, and the pain ceased. Before the wound healed, two sloughs came away in the direction of the blood-vessels, nearly three inches in length, after which cicatrization proceeded rapidly. But great pain, referred to the lost hand, continued, and the patient felt as if the left hand was still attached, and in a high state of inflammation, with the fingers rigid and immoveable. There was also a sense of pain in the region of the cervical vertebræ, with distressing headaches, especially when any acidity prevailed in the stomach. Nevertheless the appetite continued excellent. With the view of getting rid of these painful sensations, the patient visited Edinburgh, London, and Paris, where he consulted the most eminent of the faculty in each capital. The majority of these recommending a second amputation of the arm, as the most effectual means of relief, the operation was performed by Mr. Wardrop, between three and four years ago, in London. His evil fate pursued him from North to South! In about twelve hours after the operation, hæmorrhage took place, and Mr. W. was obliged to open the wound to search for the bleeding vessel. This opening of the stump gave him more pain than all the operations he had previously undergone! The stump, after this, healed very quickly; (the amputation was performed five or six inches above the elbow) but, alas! the original sensation referred to the lost hand remains as intense as ever! Since the last operation, the patient has been gradually losing ground, and, with the exception of a good appetite, he is suffering under all the usual symptoms of dyspepsia. In addition to the pain referred to the absent hand, the patient is harrassed with constant spasms in the biceps and other muscles of the arm, and subsultus. There is also a continual tinnitus in the left ear. He takes a good deal of exercise, but still his sufferings are on the increase. Every thing he eats is disposed to turn acid, and when acidity obtains in the stomach, all the above-mentioned phenomena are greatly exasperated. "I find there is a small hardened portion of substance on the face of the stump, connected with the muscles, very painful on pressure, (the sensation always referred, even then, to the lost hand) and which appears to me to be a matting of nerves."

Previously to the last (second) amputation, Sir Astley Cooper advised merely the removal of the extremities of the nerves on the face of the stump. Mr. Abernethy was averse to any operation, and recommended blue pill. We have ourselves consulted several eminent surgeons and physicians, but their opinions have been very various. Sir Henry Halford is of opinion that the extremities of the nerves on the face of the stump are in a state of disease, and are the cause of the patient's sufferings. He recommends another amputation, which he has known to succeed in two instances. We forbear to state any opinion on the subject, at present; but solicit the opinions of others. The patient is employing every means of improving the general health, and especially the state of the digestive organs.

It will be acknowledged that there is hardly to be found on record a more melancholy, unfortunate, or distressing case, than that which is here sketched out. As occurring in the person of one of our own profession, it is doubly interesting, and calculated to call forth our sympathy. Any suggestion communicated to the Editor of this Journal will be conveyed to the afflicted patient.

---

### 4. Observations on Diabetes. By M. Hufeland.

In a recent number of his journal, M. Hufeland relates a case of this disease cured by opium, and then appends some comments. The case was as follows:— A man previously robust, became, at the age of 45, affected with diabetes mellitus, without any other assignable cause than frequent applications of cold to the surface of the body. Various remedies were tried in vain. He was then put upon a course of opium, combined with ammonia, which he continued from May till September, by which period he was completely cured.

The narration of this case gives M. Hufeland an opportunity of enunciating his ideas respecting the nature of diabetes. His doctrine is simply this: that the proximate cause of the disease consists "in a pathological change in the secretory action of the kidneys—the effect of which change is to substitute sugar for the constituent principles of the urine —and to deprive the blood of the saccharine nutriment." We are disposed to agree with a French critic, who remarks that "this explanation, which is no explanation at all, advances us very little in the knowledge of the disease, pathological or therapeutical." The same critic makes the following remarks:—"Diabetes being a chronic disease, its remote causes may be included in the following category:—debility of the kidneys—nervous super-excitation of the spinal marrow and renal plexus—sanguineous congestion—chronic inflammation—abdominal irritation—metastasis and local disorganization. These different causes require different methods of treatment—and hence we may explain the success, and the want of success, which have attended particular remedies and modes of practice." Vous avez raison monsieur!

---

### 5. SUDDEN SENSE OF SUFFOCATION.

There are many affections—they may only be *symptoms*—for which we can find no appropriate designations in systems of nosology, but which nevertheless occasion great anxiety to the mind of the practitioner—great danger to the patient. Among these may be mentioned a sense of sudden suffocation which comes on people, generally when in bed, without there being evidence of any organic or other disease in the heart or lungs. Thus, we are acquainted with an elderly gentleman, who has long been a martyr to gout, and on whose joints are numerous chalk-stones. His heart and lungs present no appearance of disease, as far as these functions are concerned, or as auscultation and percussion can determine. At uncertain periods, and usually in the night, he is seized with such a sense of suffocation, that his life appears, and we believe is, in the greatest jeopardy. The loss of four or five ounces of blood gives instant and entire relief from this terrible state of strangulation. In the intervals his breathing and circulation are free. He has taken Wilson's tincture for the gout during the last ten years. When no person is at hand to open a vein, this gentleman has a long and desperate struggle with the spasm, and derives little relief from any medicine till blood can be drawn. This case was brought to our recollection by the perusal of one published by the younger Dr. Lacheze, Professor of Medicine at Angers.

*Case.* On the 20th February, at eight o'clock in the evening, Madam ———, being in the midst of her family, and having no previous symptom of disorder, felt, all at once, such a sense of suffocation that she could scarcely get in the smallest quantity of air into the lungs. In twenty minutes from the commencement of the attack, Professor Lacheze was with the patient, and found her in the following condition:—extreme agitation—dreadful dyspnœa—countenance somewhat flushed, and indicative of great distress—the inspirations at long intervals—pulse concentrated and a little accelerated—occasional paroxysms of cough, without expectoration—the patient thought her whole complaint was in the left side of the chest. As the lady appeared on the very brink of the grave, Dr. L. instantly opened a vein, and the blood had scarcely begun to flow, when relief was obtained. She said that she felt as if a load of one hundred pounds were taken off her chest. Only eight or ten ounces were taken away, and the patient was quite well. Two years previously, she had experienced a similar attack, and was relieved by similar means. —BIBLIOTHEQUE MED.

As these paroxysms are not unfrequently connected with a gouty diathesis, if not dependent on a gouty cause, no more blood should be taken than is just sufficient to relieve the symptoms; otherwise the malady may be rendered worse instead of better by the depletion. These sudden attacks, we conceive, are not to be classed under the head of asthma. They come in an instant—they are as quickly removed—and the remedies are not the same.

---

6. TRUE PHILOSOPHY—OPPOSITION.

At a dinner given by the Phrenological Society of Edinburgh to Dr. Spurzheim, on the 25th January last, there was some excellent speaking; and, among the *appropriate* toasts usual at all public dinners, we perceived ONE, given by Mr. Scott, which deserves commemoration, as marking the progress of liberality and true philosophy in a very striking manner. It was received with rapturous applause, and drunk with "three times three." It was—"THE OPPONENTS OF PHRENOLOGY!" When one of Shakspeare's clowns was asked "how he did?" —his answer was, "*the better for my foes, and the worse for my friends.*" So, we are convinced, will it be generally found in this world. From friends comes FLATTERY, the most dangerous as well as the most delicious unction that can be laid to the soul! They praise us for qualities which perhaps we do not possess—or magnify those virtues or acquirements which we have. At all events, we may be assured that, next to a good friend, is the advantage of having a stanch enemy—one who deals not in soft blows and petty skirmishes, but one who enters the field in earnest, and warns you that he will *take* all advantages, and *give* no quarter. This is the enemy which we prefer. We quite agree with Mr. Scott, that—"opposition generally is a good thing, and is the means of producing good in every case where it is duly applied. It stirs up the languid faculties, and rouses them to exertions which they would not otherwise make. No science, art, invention, or discovery of any kind, has ever been perfected, without first running the gauntlet of a hot and determined opposition." The good effects of opposition indeed are seen in every thing, from the national councils down to rival green-grocers. In literature and science —even in medical science, opposition is the soul of industry, the patron of talent, the sun that draws forth the flowers of genius. It is on this account, that we rejoice at the prospect of two universities in this metropolis. The one will be a constant whetstone to the other. Had it not been for the first, the second would never have sprung into existence—and if no *second* had come forth, the professors of the *first* would have laboured under the greatest of all misfortunes—*the want of an opponent.* For our own parts, we are, or ought to be, more than usually grateful to Fortune; for Heaven has blessed us with a preternatural lot of bitter enemies—the best friends we ever had. May they live long and multiply fast!

---

7. CURIOUS INSTANCES OF PARTIAL AGUE.

However strangely the above title may sound in some people's ears, we have no doubt that the causes which produce intermittent and remittent fevers will sometimes—nay, very often, act on a single region, or even a single spot of the body, and there induce a train of symptoms which are little suspected of aguish character. When we have stated the following facts, these opinions will not seem so chimerical.

Madame B———, aged 22 years, and previously healthy, was suckling her infant at the time that the following attack came on without any known cause. She was seized with a cold chill, which was succeeded by heat and cephalalgia of one side of the head and face, being exactly the left half of these parts. After a few hours' duration, these symptoms disappeared in a perspiration of the parts above-mentioned, and of those only. The next day, and at the same hour, another and a similar attack took place, and was much more severe. The paroxysm terminated by perspiration as before, and after an equal duration. A physician, Dr. Lassalvy, was witness of the third paroxysm. After a rigor, which commenced in the back, and radiated to the upper and lower extremities of the left side, lasting a quarter of an hour, a reaction took place in the same side, with cephalalgia, flushing of the left cheek, glistening of that eye, lachrymation, pain of the scalp on pressure, and slight convulsive movements of the left eye-lid and side of the upper lip, redness and dryness of one side of the tongue. The face now presented a very curious spectacle—red, tense, and shining on the left side, while the right was perfectly natural. Both sides of the chest sounded well; but in the left side, the "râle crepitant" was distinctly heard, without cough or expectoration. The left side of the epigastrium and of the abdomen was painful on pressure. The attack commenced at 8 o'clock, and increased till 10, when it began to diminish, and finally disappeared in a perspiration on the side affected. The sulphate of quinine was administered—the fourth accession was mitigated, and the whole of the phenomena soon disappeared.

Cases analogous to the above may be found in many authors. M. Duval states (*Obs. Med. Chemic.*) the case of a soldier in the Military Hospital of Brussels, who was affected with a quartan ague presenting the following anomaly. The cold fit, which generally lasted two hours, was accompanied by violent muscular spasms of one side of the body, which gave the face a most hideous appearance. What was still more remarkable, too, the right and left sides were alternately affected in each succeeding paroxysm.

In the REVUE MEDICALE for 1814, we have the case of a man who slept in a room newly painted, and who was seized with an intermittent fever, which only affected that side which was next the painted wall of the room where he lay. Mangold (Erfurt, 1766) relates an instance of quartan ague, accompanied by hemiplegia, perspiration, and convulsions, sometimes in the right, sometimes in left half of the body; but never in both at the same time. M. D'Auxeron (*Journ. de Med.* t. xxiv.) details a case where ague affected the lower half of the body only—from the diaphragm downwards. VANSWIETEN has recorded a case of *catalepsy* affecting only one side of the body. Morgagni instances a case where icterus was confined to one side—that side being paralytic. He states that the boundary between the jaundiced and unjaundiced sides were remarkably distinct, the two sides of the face, and even of the nose, presenting a most singular contrast. Etmuller, Paulinus, Plenck, and others, have put similar cases on record.—JOURNAL DE PROGRÈS, Vol. VIII. 1828.

We have dwelt the longer on these facts, because diseases of an aguish character, that is, of malarious origin, are now extremely prevalent throughout England, assuming every form of Proteus, every colour of the rainbow—and imitating half the diseases in the most complicated systems of pathology. The facts and arguments which we have brought forward from Dr. Macculloch's work, will receive, we strongly suspect, a great accession of strength from the "atmospheric constitution," as Sydenham would call it, now prevailing over these islands, and indeed over the Continent generally. Were we inclined to speculate, we would hazard an opinion that the epidemic cholera of India, which, after making various circuits in that extensive quarter of the globe, began to travel westward, and actually reached the borders of the Black Sea, is now actually in Europe—not indeed in its frightful oriental attire—but under a variety of masked forms, that are chiefly characterised and detected by one general feature of periodicity, intermission, or remission. The *detection*, however, requires a minuter examination than most practitioners, in the hurry of business, are inclined to bestow on what are considered as only modifications or anomalies of the common diseases of the time and place. It is on this account that we intreat them to direct their attention to the prevailing disorders of the present period, being well convinced that they will soon perceive a very peculiar character in the majority of those that are thus narrowly investigated.

---

### 8. NEW METHOD OF ACCELERATING THE SEPARATION OF THE PLACENTA FROM THE UTERUS. By DR. DUPARQUE.

We have, on a former occasion, made our readers acquainted with Professor Mojon's proposal and practice of arresting uterine hæmorrhage, by injection of cold water into the vessels of the placenta—and also his proposal of injecting solution of chloride of lime, through the same channel, in cases of retention of the secundines, in order to destroy the putrid miasms generated by the corrupting mass. Taking a hint from these proposals, Dr. Duparque has put into practice, in two cases, the injection of cold water through the channel of the umbilical veins, in order to induce uterine contraction in cases of retained placenta. In both instances, the patients complained of pain in the loins, as soon as the injection reached the placenta, and strong uterine contraction was the immediate consequence, with separation and ejection of the placenta. The injection is easily performed by means of a common syringe—the practice cannot be attended with the slightest danger—and it may, perhaps, be preferable to much traction by the cord, or the forcible introduction of the hand.—BIBLIOT. MED.

9. MELANCHOLY INSTANCE OF GRADUAL ABOLITION OF ALL THE SENSES IN SUCCESSION, THE INTELLECTUAL FACULTIES REMAINING ENTIRE.

Those who are accustomed to visit the asylums of the insane, not unfrequently witness that dreadful death by piecemeal, which is preceded, if not occasioned, by a gradual and successive abolition of all the senses by which we communicate with the world around us. Fortunately, in all these cases, or almost all, the intellectual faculties have previously become so deranged, that the loss of the external senses is little, if at all, felt. Not so in the following instance, where proud man might be almost said to be cursed with reason, and with consciousness, alas! too keen, of his living death!

*Case.* The patient was evidently a man of some rank and consequence during the reign of Napoleon; and held an important post in the financial department of the government. He was a man of wit—a lover of the fine arts, and a devotee of pleasure, which had been abused. All at once he became affected with gutta serena, and was left, like Milton, in total darkness. Having suffered in his youth from syphilis, he was put on a course of mercury, without any benefit. Afterwards he tried numerous remedies under the best physicians in France, but to no purpose. Notwithstanding this blindness, he continued to fulfil his functions as financier, and acquired such a tact that he could distinguish engravings on copper from woodcuts, lithographs, &c. by the fingers alone. In short, he enjoyed society nearly as much as ever, and hardly felt the loss of sight. But in a few years he began to grow deaf of one ear—then of the other, and in a short time, he had to add the total loss of hearing to that of sight! By means of large moveable types or letters, which his family put together, he was soon able to read with his fingers whatever was wished to be communicated, and by this contrivance he still held free intercourse with the external world. All his intellectual faculties remained unimpaired, and his memory was extremely tenacious. But new misfortunes were in store. Muscular motion and sensibility began to fail, and, in a short time, they were completely extinct! He was now, as it were, exiled from the earth in the midst of his family and friends! He could speak; but no answer, no sign, no impression could he receive through any channel of sense! In this deplorable condition, it was accidentally discovered that a small portion of one of his cheeks retained its sensibility, and the active imagination of the sufferer soon took advantage of the discovery. He caused one of his sons to trace letters on his cheeks as he dictated them, and by constant repetition he was soon able to recognize these letters as traced on the sensible part. He made such progress that, in a few days, his son wrote on his father's cheek the speech of the King of France on his return in 1815, the whole of which was completely understood! With this sole solace of a dreary death in life, he dragged out some time, in a state of the greatest misery that can well be imagined—his intellectual faculties not appearing to suffer the slightest degree of decay. At length the *involuntary* muscles, or those of the organic life, began to partake of the general paralysis of the locomotive muscles—and vitality at last ebbed and finally disappeared!

The above case, which is published by M. Defermon, affords a remarkable example of abolition of function in all those portions of brain to which the nerves of sense and of motion lead, without any affection of those other portions in which the intellectual faculties reside, or through which they are manifested! The case offers food for profound reflection.

10. FATAL ICTERUS.

When jaundice continues for any length of time, without the common symptoms of gall-stones, we generally suspect some change of structure in the liver itself—or some tumour pressing on the ducts. The following case is interesting in several points of view.

A man 56 years of age, became affected with jaundice, which lasted several months, when he died. No accurate history, however, of the symptoms could be obtained, but the body was very accurately examined in the Civil Hospital of Nancy, and this was the report. Almost all the interior parts, solids and fluids, were tinged yellow, and, on chemical examination, they exhibited proofs of bile. The pylorus was sound. Two or three inches from the origin of the duodenum, there was a tumour with elevated edges, rather larger than a crown-piece, the surface of which was uneven, very hard to the touch, of a whitish yellow colour, and very vascular. The different coats of the intestines were involved in the disease, which included, at one of its edges, the openings of the biliary and pancreatic ducts, which were extremely contracted, and almost annihilated. When this tumour was incised, it was found to be about half an inch in thickness—hard, and, in short, complete scirrhus. The liver was larger and heavier than natural, and of a brownish red colour. The gall-bladder was extremely distended, and when strongly pressed, a few drops of bile issued forth into the duodenum. All the three ducts, cystic, hepatic, and common, were enormously dilated—so that two fingers could be introduced into the ductus communis, when cut across. The fluid in the liver and ducts had little resemblance to healthy bile.—JOURNAL DE PROGRES.

It would have been very interesting to have had a history of the symptoms in this case, since it was one where death, in all probability, was caused by the absence of bile in the alimentary canal, and its presence in all the other parts of the body. It can hardly be supposed that death was caused by the scirrhous tumour in the duodenum, as it had not come to a state of open cancer, and was not so situated as to interfere materially with any other function than that of the liver.

---

### 11. DREADFUL INSTANCE OF INFANTICIDAL MONOMANIA.

In a small town near Copenhagen, a horrible case of the above has recently occurred, the particulars of which are published by Dr. Otto, in the last (17th) number of the PHRENOLOGICAL JOURNAL. The unhappy criminal (if such, a monomaniac can be termed) is a Peter Nielsen, aged 47 years, the father of seven children, of which he drowned, at one time, FOUR! He appears to have experienced some misfortunes, but was not in positive want of the necessaries of life at the moment when he committed the horrid deed. It is evident, from the testimony of several witnesses, who conversed with him, both before and after the transaction, that he was not intoxicated, nor in the least agitation of mind. He was, on the contrary, placid and tranquil. No domestic altercations, of any moment, had occurred—but he was disconcerted at not readily getting a new lodging on being turned out of that which he previously inhabited. In short, no external or obvious motive to this terrible act of infanticide could be traced. He evinced no disposition to melancholy, "nor had he read any mischievous books." His love to his children was testified by all his acquaintance. He confessed that the idea of killing his children came into his head on the morning of the day that he put the idea into execution—and that the impulse was quite irresistible. He determined to drown the three younger boys, and to spare the daughter, who was older. But the unfortunate girl insisted on accompanying her father and brothers in the walk which he proposed, and though he endeavoured to persuade her to return, she would not. He averred that his motive for destroying the children was the fear of not being able to maintain them. He wished to spare the girl—not because he loved her more, but because she had made more progress, and was better able to provide for herself. Having arrived at a pond, he first embraced his children, and then hurled them all into the water! He stood by unmoved, and saw them struggle and sink! He then returned quietly to the town, and there disclosed the whole affair. He was conducted back to the pond, and saw the dead bodies of his children, without evincing any emotion of mind. For a moment he wept, when he saw the bodies opened (for the purpose of medico-legal proof of the kind of death) but soon regained his tranquillity. He affirmed that he did not destroy his offspring in order to procure happiness for them in Heaven, nor from any desire to be put to death himself, as he wished to live.

We think every enlightened mind will view this case as one of monomania. Some ungovernable impulse, without rational purpose,—some hallucination of reason, must have urged him to the unnatural act. We agree with Dr. Otto, that this infanticidal impulse by no means proves that the parent was originally or naturally deficient in the love of his offspring. "Every faculty, even the strongest, may be overwhelmed and overcome by a strong passion—or insanity in another faculty." How many honest and moral men do we see, in the hour of temptation, yield to this or that passion? Dr. Otto endeavours to account for the physical cause of this monomania, by supposing that a temporary congestion of blood, or excitement, takes place in a portion of the brain, for instance, in the organ of DESTRUCTIVENESS—thus causing an involuntary or invincible propensity to kill. In whatever way we account for the *cause*, we are convinced that monomania existed at the time when this infanticide was committed, and that the verdict should be accordingly.

---

12. A CASE ILLUSTRATIVE OF THE EFFECTS OF A DIVISION OF THE SPINAL MARROW BETWEEN THE THIRD AND FOURTH DORSAL VERTEBRÆ, IN THE HUMAN SUBJECT, WITH REMARKS. By WILLIAM WALLACE, M.R.I.A., &c.

[Dublin Transactions, Vol. V.]

A man servant, aged 44, fell from a drawing room window into the area, on the 11th Decr. 1826. Mr. Wallace found him supported on a chair, and crying lamentably on account of violent pain in his back. His countenance was pale and anxious—surface cold—eyelids closed—pupils dilated—pulse small and weak. This was in 15 minutes after the fall. He was removed to bed, and seemed to have no muscular power, or consciousness. An obscure crepitus was felt among the spinous processes between the scapulæ—and pressure there seemed to occasion intolerable pain. There was no external mark of violence on the back. He could not, or would not answer questions—but continued to cry out with pain. He was conveyed cautiously to the Jervis street Infirmary, and at 8 in the evening, Mr. W. again visited him. He was now comparatively tranquil—pulse small and weak—respiration scarcely audible—appeared stupid and inattentive; but, when roused, he answered questions distinctly. Still complained of great pain in his back, between the scapulæ—had no sense or motion of the lower half of the body. The catheter was introduced, and the urine drawn off, and directions were given to watch the approach of re-action, and then to bleed.

12th. The re-action took place at 11 o'clock last night, when twelve ounces of blood were abstracted, and an enema thrown up, which returned without any fæces. He spent a restless night, with sickness of stomach, and now appeared in great agony—pulse above 100, and bounding—breathing quick and oppressed—great irritability of stomach—abdomen full and tense—no discharge of urine or fæces, except what of the former had been drawn off yesterday by the catheter. "No sensibility or motility from the seventh vertebro-sternal rib downwards." The line of demarcation between the sensible and insensible parts was very accurately defined. The water drawn off was ammoniacal—8 ounces of blood were taken from the arm—purgatives ordered, but were quickly rejected from the stomach, the enema returning without effect. Nothing would tranquillize the stomach. 13th. Continues much the same—nothing will lie on the stomach—no evacuation from the bowels. There was no sense of nausea attending the vomiting.

Some tartrite of antimony was dissolved in a large quantity of whey—a grain to the pint—and he was desired to drink freely of the solution. He was not, however, expected to live through the night. 14th. To Mr. Wallace's astonishment, the patient was much better this morning. His countenance was revived—vomiting and hiccup had ceased—abdomen softer and much less full—thirst unabated—tongue white and dry—pulse slower and fuller. He had a sleep in the night, after which, the bowels had acted freely. He had taken 4 grains of the tartrite, in the course of the night, in four pints of whey. This last was ordered to be continued for common drink, with the same proportion of tartrite as before.

15th. Took 4 pints of the emetic whey during the last 24 hours—no sickness—bowels open. Complains much of palpitation of the heart, with soreness of the chest, and inability to cough. In the evening of this day, the patient seemed very much better than at any period since the accident occurred. The thirst was trifling, the tongue cleaning, the pulse decreasing in frequency, the abdomen softer, the respiration natural, the countenance tranquil,—in short, all the symptoms improved—but still the paralysis of the lower part of the body remaining.

16th. This morning there was an alarming change for the worse. He lingered out, however, till the 19th, that is, till the ninth day of the accident, when he expired, with most of the symptoms of typhoid fever.

*Dissection nine hours after death.* The muscles were universally rigid. By means of a small saw the spinous processes of the vertebræ were separated from the transverse processes, and removed, so as to expose the vertebral canal and its contents.

"The spinous processes of the second, third, and fourth dorsal vertebræ were fractured at their root. The ligamenta subflava and interspinalia belonging to the fifth and sixth vertebræ were torn. A large quantity of dark blood was effused into the spinal canal, round the theca vertebralis, insulating the latter from the former, and extending from the upper to the lower end of the spine. The theca vertebralis was lacerated on its dorsal aspect to the extent of three-eighths of an inch, opposite to the third vertebra of the back.

"The spinal marrow, with its proper membranes, was torn across at that part where it corresponds to the interval between the third and fourth dorsal vertebræ. Its lacerated ends were separated to the extent of half an inch, and the interval filled with blood.

"The vaginal ligament was lacerated at the interval between the third and fourth dorsal vertebræ, and blood was effused between the posterior surfaces of the bodies of the vertebræ and this ligament. The intervertebral substance, which corresponded to the rupture in the vaginal ligament, was torn from the vertebra above. The spine, viewed from the thorax, did not present any marks of injury." 213.

The vessels of the head were very much congested, and a thin stratum of effused blood covered the posterior, the superior, and the external surfaces of the posterior lobes of the brain. This effusion was between the pia mater and the surface of the brain, and was extremely thin, like the impression of a camel's hair pencil charged with blood. The substance of the brain was firm, and there was a small quantity of serum in the ventricles. The internal surface of the stomach was very vascular, and much of the small intestines was nearly black, with extensive intus-susceptions. The liver was firm—the gall-bladder contained a very small quantity of viscid fluid, resembling the white of an egg. The whole system of the vena portæ was greatly distended with very black blood. The veins on the outer and inner surface of the pleura were minutely injected with dark coloured blood, so as to form a beautiful vascular net-work, rendering the membrane nearly black. The surfaces of the pleura were glued together by a very thin stratum of adhesive matter. The lungs were firm, and filled the cavity of the chest. They were of a dark colour, and gorged with black blood. The cavities of the pleura contained a few ounces of dark serous fluid. There were three ounces of yellow serum in the pericardium. The heart was large, and distended by black blood, partly coagulated.

*Remarks.* The observation of Nysten is confirmed by this case—namely, that division of the spinal marrow does not prevent or diminish that rigidity of the muscular system, which is, perhaps, the result of its last vital action. The phenomena presented on dissection render it probable that the blood did not undergo the proper change in the lungs, and that there was an undue accumulation of blood in the right side of the heart thereby occasioned. Serous effusions were the natural results. There is reason to infer, that the biliary secretion was suspended. The ammoniacal state of the urine is the usual consequence of the spinal accident; and inflammation of the bladder is often the cause of death. We may here remark, that in cases of paraplegia from spinal injury, the introduction of the catheter is frequently productive of great mischief. The parts are insensible ; and the inexperienced house-surgeon (when the patient is in hospital) works away, and thinks he is doing no harm while the patient does not complain. The effects appear, sooner or later, in the shape of inflammation or sloughing of the urinary passages. Mr. Bell has noticed this, among many other excellent observations of that talented surgeon.

"Was the discharge from the bowels on the fourth day the result of the action of the tartar emetic, or was it the consequence of the injury of the spinal marrow? When a bladder has been paralyzed from injury of its nerves, retention of urine is the immediate result, but sooner or later this is followed by incontinence, as was the case in the present instance. This phenomenon is to be explained by a law to which the action of nerves appears to be subject, viz. one degree of pressure or irritation will produce spasm or convulsion, and a greater degree of the same will cause loss of power, or paralysis. This law will be found to govern the muscular system of both voluntary and involuntary action, and consequently the sphincters." 219.

It need not excite surprise, in this period of surgical science, that the patient should have lived so long after complete division of the spinal marrow. Independent of Magendie's celebrated case, where a considerable portion of spinal marrow was found obliterated, and yet where sensation and motion in the lower extremeties remained, we believe that Mr. Bell met with a case of complete division of the "spinal brain," and where the patient lived nearly a year;—and died, at last, of some eruptive disease—and not at all of the spinal injury. It is more than probable, that had Mr. Bell's patient not died of another disease, a considerable degree of sensation and motion would have returned—by a kind of collateral circulation of the nervous energy, in the same way that the circulation of the blood is carried on by circuitous routes, when the regular channel is blocked up.

---

### HOSPITAL PRACTICE.

### ST. GEORGE'S HOSPITAL.

### 13. INJURIES OF THE HEAD.

Sir Astley Cooper was the first to draw a distinction between simple and compound fracture of the skull, a distinction on which is hinged a very material difference of treatment. It has been argued, that this distinction is imaginary, and that there is no analogy whatever between fractures of the cranium and fractures of the long bones. The objection is more specious than solid. If there be a compound fracture of the femur, will any one be so bold as to affirm, that the danger which ensues is merely dependent on the injury of the *bone?* Does the patient die of caries of the *bone*, of necrosis, or exfoliation, or rather, when he sinks, is it not by reason of the mischief to the soft parts, the blood-vessels, the nerves, the muscles, or the tendons? Are the wasting suppurations which so frequently destroy him, in the bone or the parts around? Most undoubtedly the latter; though, of course, they are aggravated by the injured condition of the former.

In fractures of the cranium, the mischief is not in the parts without, but those within—the meninges, or the brain itself. The membranes of the brain are serous membranes, and extremely prone to inflammation, whilst they are as carefully excluded by nature from the contact of the air, as the parts around the femur, the tibia, or any bone whatever. The same thing holds with the brain itself, and yet there are some who deny that it can make the slightest difference, in fractures of the cranium, whether the membranes or the brain are exposed to the atmospheric influence, or not.
